# Analyses of Total Alkaloid Extract of* Corydalis yanhusuo* by Comprehensive RP × RP Liquid Chromatography with pH Difference

**DOI:** 10.1155/2016/9752735

**Published:** 2016-09-19

**Authors:** Xiaodong Wei, Hongling Shen, Lijun Wang, Qingyan Meng, Wenjie Liu

**Affiliations:** ^1^College of Life Science, Tarim University, Alar, Xinjiang 843300, China; ^2^Key Laboratory of Protection and Utilization of Biological Resources in Tarim Basin of Xinjiang Production and Construction Corps, Alar, Xinjiang 843300, China

## Abstract

A comprehensive two-dimensional (2D) reverse phase (RP) liquid chromatography (LC) method is developed for alkaloid analysis. This offline comprehensive 2D method is developed using different pH values. With a pH value of 10.5, most alkaloids appear in the form of neutral molecules possessing high retention factors based on their polarity, while the alkaloid polarity order is changed when the pH value decreased to 3.0. The performance of pH modulated 2D LC is demonstrated with 8 alkaloid standards which resulted in orthogonal separation. The developed method is then applied to total alkaloid separation in* Corydalis yanhusuo*. The first-dimension separation is carried out using methanol and water containing 1.0% ammonium hydroxide and a strong base-resistant RP column, which afforded a peak capacity of 94. The second-dimension analysis is carried out with a surface positive charge column providing a peak capacity of 205 using a mobile phase consisting of acetonitrile and water with 0.15% formic acid. 2D analyses of total alkaloid extract from* C. yanhusuo* afford a total peak capacity of 9090. Sixteen compounds were tentatively identified based on their ultraviolet spectrum and MS/MS analyses. The proposed method provides an alternative approach to achieve high peak capacity for analysis of alkaloid extract.

## 1. Introduction

Alkaloids belong to a group of naturally occurring compounds that contain one or more basic nitrogen atoms [1]. Alkaloids can be produced by a large variety of living organisms such as bacteria, fungi, plants, and animals [2]. Alkaloids play an important role for living organisms and are assumed to have protection functions for plants. For example, some toxic alkaloids such as swainsonine originally found in* Swainsona canescens* prevent animals and insects from eating it [3, 4]. Most alkaloids evoke a bitter taste that accounts for the unpleasant taste of some traditional herbal medicines.

The most outstanding and attractive property of alkaloids is that they may have a wide range of pharmacological activities. Many alkaloids such as ephedrine [5], quinine [6, 7], camptothecin [8], and opioids have been intensively used in traditional herbal medicines as well as modern medicines to treat asthma, malaria, cancer, acute pain, and other diseases. Some alkaloids found in medicinal plants appeared as leading compounds for drug discovery. For example, huperzine A comes from a type of moss that grows in China,* Huperzia serrata*, and has been used as a reversible acetylcholinesterase inhibitor and NMDA receptor antagonist that crosses the blood-brain barrier and was shown to be an efficacious medicine to treat Alzheimer's disease [9, 10]. For the limited natural resources, the synthesis of huperzine A analogs appeared to be an attractive field and some have shown higher activities compared to huperzine A [11–13]. Other alkaloids, including cocaine, caffeine, and nicotine, possess restorative or stimulating activities and have been used in energy drinks or as recreational drugs. Some alkaloids can be toxic to human beings such as atropine and solanine [14, 15]. The seeking of new bioactive alkaloids from plant extracts appeared to be an attractive source for drug discovery [16]. Though opioids play important roles in the treatment of acute and chronic pain, the search for novel analgesics is an urgent task for the side effects and potential of drug addiction caused by opioids. Recently, dehydrocorybulbine, an analgesic alkaloid compound isolated from traditional Chinese medicine* Corydalis yanhusuo*, showed a surprising mechanism of inhibitory action against dopamine D2 receptor in clinical trials [17]. Discovering of new alkaloids from* C. yanhusuo *extract has aroused great interests.

Various analytical methods have been used for the determination of alkaloid extracts of medicinal plants like gas chromatography [18], gas chromatography mass spectrometry [19], electrophoresis [20], liquid chromatography, and liquid chromatography with mass detection, along with other techniques [21–23]. However, the seeking of novel analytical methods is necessary for the diversity of alkaloids and the complexity of samples, especially for the discovery of low abundant alkaloids and structurally similar alkaloids. Comprehensive two-dimensional liquid chromatography (LC × LC) provides high peak capacity which leads to significantly improved analytical performance compared to conventional single-column liquid chromatography by using orthogonal separation mechanisms [24–27]. For the separation of alkaloids, the combination of 2-dimensional separation generally consists of strong cation exchange (SCE), size exclusion chromatography (SEC), and hydrophilic interaction liquid chromatography (HILIC) with reverse phase (RP) chromatograph [28]. Nevertheless, SCE, SEC, and HILIC mode have limited separation capacity compared to RP chromatography, which decreased the total peak capacity of two-dimensional separation. The incompatibility of the mobile phase of two dimensions also restricted the application of those combinations. RP × RP with different column also showed improved performance versus single dimensional separation [29]; the correlation between the two dimensions showed a low degree of orthogonality. Fortunately, the orthogonality between the two RP dimensions is greatly improved by using different pH values for the separation of proteomic samples and carboxylic acids [30]. The applications of different pH values on the first and second dimensions for 2D LC separation have been reported in the literature for natural products [31], peptides, and pharmaceutical samples [32]; however, the analytical application to alkaloids still needs to be detailed.

The objective of this paper is to develop a LC × LC method for the separation of total alkaloids extract using different pH values to achieve a high degree of separation orthogonality and high peak capacity. Traditionally, the separation of alkaloids with HPLC demands a basic buffer to decrease serious tailing by minimizing the interaction between the basic analytes and acidic residual silanol groups of the column material, or it improves performance by ion-pair chromatography [33]. With positive charged surface particle column [34], it is possible to achieve high efficacy with low ionic strength mobile phases for the separation of basic compounds. More important of all, the elution order of alkaloids is significantly influenced by the pH value of the mobile phases. The performance of the proposed pH modulated RP × RP comprehensive two-dimensional liquid chromatography, including orthogonality and peak capacity [31], was evaluated using eight alkaloids standards and the total alkaloids of* C. yanhusuo*, a traditional Chinese medicine to alleviate neuropathic pain.

## 2. Experimental

### 2.1. Chemicals and Reagents

All HPLC grade solvents were obtained from Sigma-Aldrich (Buchs, Switzerland) and filtered with 0.22 *µ*m membrane before using. Formic acid was purchased from J. T. Baker (Phillipsburg, USA). Analytical grade 99% ethanol and ammonium hydroxide were provided by Sinopharm Chemical Reagent Co. Ltd. (Shanghai, China). Eight alkaloids standards, that is, oxymatrine, cytosine, hordenine, sophoridine, sophocarpine, matrine, evodiamine, and rutecarpine, were purchased from TCI (Shanghai, China). Water was processed with a Milli-Q ultrapure water system (Millipore, Italy). The root of* C. yanhusuo* was purchased from a local traditional Chinese pharmaceutical store and identified by Professor Yuan Liu from the Department of Chemistry in Southwest University for Nationalities in China.

### 2.2. Sample Preparation

One hundred grams of whole dry root of* C. yanhusuo* was ground with a home homogenizer and then extracted three times with 2.4 liters of 60% ethanol for 1 hour in an ultrasonic bath (Kunshan, China) under ambient temperature. The extracts were combined and filtrated under vacuum. The filtrate was then evaporated to dryness with rotary vaporization at 60°C under reduced pressure. The residue oily solid was then suspended in 1.5 liters of 0.01 M hydrochloric acid. After filtration, the pH of the solution was adjusted to 12 carefully with 1 M NaOH, and then extraction was performed with 1.5 liters of ethyl acetate three times. The ethyl acetate fractions were then combined and concentrated to dryness with rotary vaporization at 60°C under reduced pressure to afford 920 mg of total alkaloids extract from* C. yanhusuo*. Ten milligrams of solid was dissolved in 1 mL of methanol and filtered with 0.45 *µ*m membrane and used for HPLC analysis without further preparation.

### 2.3. High Performance Liquid Chromatography

Shimadzu LC AT-20 high performance liquid chromatography with dual solvent pump high-pressure gradient system, SPD-20A photodiode array detector, and an autosampler were used for the first-dimension separation. Samples were separated both on a 250 mm × 4.6 mm, 5 *µ*m particle, Waters xBridge RP C18 column with a guard column (Waters, Milford, MA, USA), and a 150 mm × 4.6 mm, 5 *µ*m particle, Acchrom PCP C18 column [34] (Acchrom, Wenling, Zhejiang, China).

Chromatographic elution under pH 10.5 was conducted with binary mobile phase gradient consisting of water (A) and methanol (B) with 1% of ammonium hydroxide in both solvents. Initial gradient conditions were set to 5% B at the flow rate of 1.0 mL/min before incorporating a linear gradient increasing to 100% B over 35 min and held for 5 min. At 41 min, the gradient was returned to the initial conditions and equilibrium for 5 min. The column temperature was maintained at 40°C during the whole process.

The chromatographic elution under pH 3.0 was conducted with binary mobile phase gradient consisting of water (A) and acetonitrile (B) with 0.1% of formic acid in both solvents. Initial gradient conditions were set to 3% B at the flow rate of 1.0 mL/min before incorporating a linear gradient increasing to 100% B over 15 min and held for 5 min. At 26 min, the gradient was returned to the initial conditions and equilibrium for 5 min. The column temperature was maintained at 40°C during the whole process.

### 2.4. Offline RP × RP Two-Dimensional Liquid Chromatography Analysis

For offline RP × RP 2D LC analyses, the first-dimension separation was performed using xBridge column under basic conditions, and each fraction of 30 seconds (0.5 mL each) of the first-dimensional separation effluent was collected to a 1.5 mL Eppendorf centrifuge tube. Collected fractions were then evaporated to dryness with a centrifuge concentrator under vacuum. One hundred microliters of 50 : 50 methanol with 0.1% formic acid was added to the centrifuge tube to reconstruct the sample solution and then transferred to 1.5 mL vials containing 250 *µ*L glass liner ready for the second-dimension analysis. The second-dimension RP-LC separation was performed on a PCP C18 column (150 mm × 4.6 mm i.d., 5 *μ*m particles) under acidic conditions. The binary mobile phases consisted of 0.1% (v/v) formic acid in water (A) and 0.1% (v/v) formic acid in acetonitrile with 20% water (v/v) (B). The elution gradient used was as follows: 5% B (0-1 min), 5–35% B (1–8 min), and 35–100% B (8–25 min), followed by 5 minutes of isocratic elution with 100% of B before returning to initial conditions at 30 min. The flow rate was 1 mL/min and the column was reequilibrated for 5 min before the next analysis. The column temperature was maintained at 40°C. For each analysis, 20 *μ*L of sample was used for each injection and the remaining sample was ready for HPLC-MS analysis.

### 2.5. HPLC-MS/MS Analysis

The structure identification of individual alkaloids was performed with MS/MS analysis and compared with published mass data and their UV spectrum. A Waters Acquity UPLC TQD triple quadruple mass spectrometer was used for MS/MS analysis with positive electrospray ionization mode. Fractions from the first-dimensional separation performed with pH 10.5 were dried with centrifuge concentrator and reconstituted to 100 *μ*L with 50 : 50 methanol, and 1 *μ*L was injected to the HPLC-MS/MS using the same column and chromatographic condition in the second-dimension analysis. The rate of the API gas was 1 L·min^−1^ and that of the dissolvent gas at 450°C was 1000 L·h^−1^. ESI voltage was 3.8 KV and the source temperature was 120°C, and the cone voltage was 30 V for precursor ion scan, with 30 V of collision voltage for product ion scan. The precursor ions were determined by a full scan mode with* m/z* from 200 to 650. Once precursor ions were determined, an automatic optimization process was performed to obtain the optimum conditions for product ion scan mode with helium as collision gas. The instrument was controlled and the data were acquired using MassLynx software (Waters). The MS/MS spectra and their UV spectra were used for the structure identification.

### 2.6. Data Analysis

Data was acquired with the Shimadzu LC solution software and exported to WaveMetrics Igor Pro 6.31 (Tigard, USA); two-dimensional chromatograms were obtained with Gizmo function and the peak capacities of 1D and 2D separation were calculated according to (1) and (2). Hence.

(1) $$\begin{array}{c} n_{c}=1+\frac{t_{g}}{1/n\sum_{1}^{n}w_{b}}, \end{array}$$

where *t*_*g*_ is the whole gradient time, *w*_*b*_ is the average peak width at baseline, and *n* is the number of peaks for the calculation. The performance of two-dimensional LC × LC was expressed by the finite orthogonality and sampling rates [24]:

(2) nc,2D=n1c×n2c×Fcβ,

where ^1^ *n*_*c*_ and ^2^ *n*_*c*_ are the calculated peak capacity from the LC separation under different pH conditions. *β* is the factor of undersampling rate of the first-dimension peaks. *F*_*c*_ is the fractional surface coverage of the practical 2-dimensional space which stands for the evaluation of orthogonality [29].

## 3. Results and Discussion

### 3.1. Separation of Alkaloid Standards with Different pH Conditions

**Figure 1:**
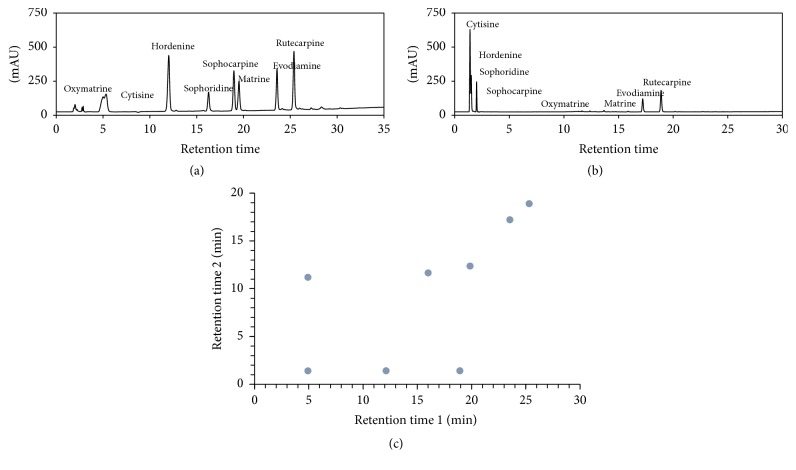
Analysis of alkaloid standards with different pH conditions. ((a) pH = 10.5; (b) pH = 3.0). (c) The scattering of alkaloid standards retention times with different pH conditions. The *x*-axis denotes the separation under pH 3.0, and the *y*-axis is the separation under pH 10.5. All chromatograms were recorded under 280 nm using a photodiode array detector.

For most alkaloids, the logarithmic association constants, pK_b_, are in the range 6~9; thus, a pH value greater than 10 provided most alkaloids with the form of unprotonated species. Accordingly, under pH value less than 4, most alkaloids are in the form of protonated ions. Consequently, the separation orthogonality of reverse phase liquid chromatography for alkaloids with different pH conditions was evaluated under pH 10.5 and pH 3.0. pH 10.5 is an extremely challenging high basic condition that is beyond the limitation of most silica-based reverse phase column materials, and the polymer-based reverse phase column material affords lower performance than the silica-based separation material in most cases; a commercially available xBridge RP C18 column with high pH stability up to 12 is selected.  Figure 1(a) shows the chromatogram of 8 alkaloid standards under pH 10.5 using methanol and water with 0.5% ammonium hydroxide in both solvents. xBridge column provided good separation for most of the 8 standards with the elution order of oxymatrine, cytosine, hordenine, sophoridine, sophocarpine, matrine, evodiamine, and rutecarpine. Under pH 3, a PCP C18 column is determined for the separation of alkaloids because of less tailing for all eight alkaloid standards.  Figure 1(b) shows the separation using acetonitrile and water as a mobile phase with 0.1% formic acid in both solvents. It is interesting to see that the elution order in pH 3.0 is completely altered compared to pH 10.5 in Figure 1(a).  Figure 1(c) demonstrates the scattering spot in two-dimensional spaces. The horizontal axis denotes the chromatographic retention time in minutes with pH 3 while the vertical axis is the retention time with pH 10.5. It is easy to conclude that the separation with two different pH conditions provides different selectivity. There is no obvious correlation with the two retention times; thus, good orthogonality is achieved with different pH conditions for alkaloid standards. It is worthy to note that the 8 standards are pairs of standards not fully resolved by both conditions; however, they are baseline resolved by another column. For example, oxymatrine and cytisine are very close in retention time and only showed little separation under pH 10.5; however, these two compounds are completely separated under pH 3 with *α* = 10. Consequently, cytisine and hordenine are almost eluted at dead time and thus overlapped under pH 3 and fully resolved under pH 10.5. Matrine, evodiamine, and rutecarpine exhibit the same elution order with two different separation conditions. Therefore, the combining of separation conditions under pH 3 and pH 10.5 would be suitable for 2D separation of alkaloids, especially for complex samples of natural products.

### 3.2. Analysis of Total Alkaloids of* C. yanhusuo *Using Different pH Conditions

**Figure 2:**
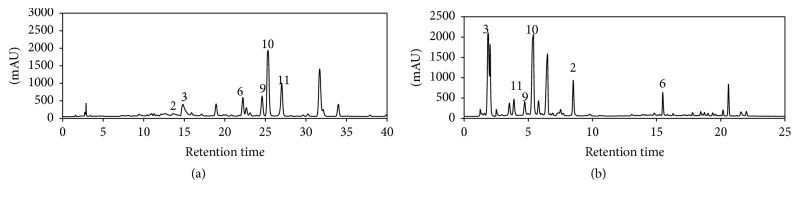
HPLC separation of* Corydalis yanhusuo* extract using different pH conditions. (a) Basic conditions using xBridge column under pH 10.5. (b) Acidic conditions using PCP C18 under pH 3.0.

The separation performance of total alkaloid extract from* C. yanhusuo* root under different pH conditions is optimized for solvents, pH modifiers, columns, and temperature. Firstly, columns with better performance including narrower peak width and lower tailing factor under acidic conditions for the separation of alkaloid extract were compared. Though xBridge column showed satisfactory performance under basic conditions, the peak shapes decreased with acidic conditions using the same column with noticeable tailing. Thus, the PCP column for the separation of total alkaloids of* C. yanhusuo* under acidic conditions was selected. Secondly, various pH modifiers including formic acid, glacial acetic acid, phosphorus acid, ammonia, phosphate buffer, and triethylamine were compared for separation capability. Though phosphorus acid afforded better peak shape and better UV detection in gradient elution over formic acid and glacial acetic acid, for the convenience of HPLC-MS/MS analysis, formic acid was finally determined as the optimum modifier under acidic conditions. Likewise, ammonia was determined as a basic modifier for volatility reason. To achieve higher separation orthogonality, methanol was chosen for the separation under basic conditions and acetonitrile was determined for the separation under acidic conditions.  Figure 2 shows the chromatogram for* C. yanhusuo *alkaloids separation under optimized conditions.

### 3.3. Two-Dimensional Separation of Total Alkaloids of* C. yanhusuo* with Comprehensive Two-Dimensional Liquid Chromatography

**Figure 3:**
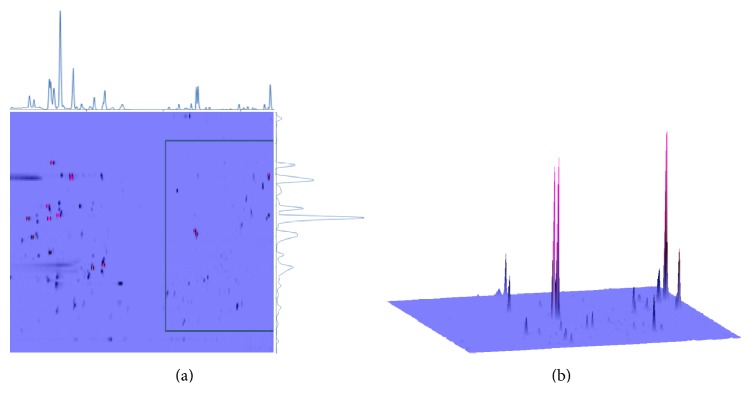
(a) A 2D contour plot of offline comprehensive two-dimensional separation of total alkaloids extract of* Corydalis yanhusuo*. The top chromatograms denote separation with pH 3.0, and the right chromatograms denote separation with pH 10.5. (b) 3D demonstration of two-dimensional separation chromatogram. The *X*-axis is the retention time with pH 3.0, the *Y*-axis is the retention time with pH 10.5, and the *Z*-axis is the absorption at 280 nm.

Comprehensive two-dimensional liquid chromatography with reversed phases in both dimensions is specifically reviewed recently for the separation of various samples including biological compounds, environmental samples, natural products, and proteomic samples, and various online schemes are discussed [27]. For the convenient reason of solvent compatibility, the offline operation method was used in this experiment. Both acidic × basic separation and basic × acidic separation were compared for orthogonality and peak capacity.  Figure 3 shows offline comprehensive two-dimensional LC × LC separation of total alkaloids of* C. yanhusuo* with both reverse phases in each dimension. Several conclusions about the LC × LC separation can be made. First, the two separation instances with different pH conditions are orthogonal to one another. That is, there is no obvious correlation between two retention times and peaks are distributed in the whole two-dimensional separation space. In some cases, compounds coelute through the chromatogram in the first dimension and are separated by the second-dimension separation. This is observed in the second dimension where several compounds are not well separated by chromatographic conditions under pH 3.0 and elute continuously throughout the two-dimensional chromatogram; they are separated by another separation under pH 10.5. Secondly, plenty of examples can be found where the number of peaks resolved in 2D space is much higher than 1D separation. For example, columbamine and canadine both have the same retention time of 15.48 min under acidic conditions; they are fully differentiated under basic conditions (22.32 min for columbamine and 29.16 min for canadine, separately). One of the most important features of reverse phase liquid chromatography is the high separation power (peak capacity). Both separation instances under pH 10.5 and pH 3.0 showed satisfactory peak capacity for total alkaloids of* C. yanhusuo*. Though several tailing peaks appeared in the PCP column dimension, the PCP column demonstrated better peak shape and narrow peak width compared to the xBridge column, which showed no tailing peak in the whole separation space. Under pH 10.5, the calculated peak capacity for the 20 selected highest peaks in 280 nm is 94 for 45-minute gradient elution. For separation under pH 3.0, 20 peaks were also selected for calculation and the peak capacity of 30-minute gradient elution was 205 instead. This high peak capacity represents satisfactory performance for a relatively short gradient time and could be attributed to the column stationary phase for its unique balance between electrostatic property and hydrophobic retention.

However, with the whole capacity for offline LC × LC separation, the peak capacity is greatly limited by the sampling time. In our experiment, each fraction was collected in half a minute; thus, the undersampling correction factor is calculated as

(3) β=1+3.35t1s×n1ct1g=2.12,

where ^1^ *t*_*s*_ and ^1^ *n*_*c*_ are the sampling time and the peak capacity for the 1D separation and ^1^ *t*_*g*_ is the total gradient elution time.

For there are no obvious correlations between the two dimensions, the surface coverage factor was estimated to be 1 approximately. Thus, the estimated peak capacity for offline LC × LC 2D separation is

(4) nc,2D=n1c×n2c×fcβ=94×205×12.12=9090.

### 3.4. Identification of Individual Alkaloids from* C. yanhusuo*

The identification of peaks from the 2D separation of total alkaloids from* C. yanhusuo* was performed manually based on their MS/MS data and UV spectra and published data. For a selected peak from the 2D spectrum, the fraction from the first-dimensional separation was selected and injected to HPLC-MS/MS analysis for the determination of the parent ions and daughter ions. It is worthy to note that the high peak capacity of LC × LC separation greatly improved the accuracy of MS detection for qualitative analysis, for there are many examples of isomers that have identical* m/z* value of their molecular ion and could not be resolved by MS spectra. In all, 272 peaks were distinguished in two-dimensional analyses, and 16 alkaloids were tentatively identified based on mass-to-charge ratio and their elution order from HPLC column (Table 1). The identified alkaloids from* C. yanhusuo* extract could be categorized into four classes, that is, protoberberine alkaloids (12), protopapaverine alkaloids (2), aporphine alkaloids (1), and protopine alkaloids (1), which stand for the most common alkaloids found in this plant. In addition, still plenty of peaks remain unidentified, especially for low abundant ones, which fully resolved from each other. It is worth noting that there are only 8 and 7 compounds that could be completely separated by either acidic or basic conditions alone.

## 4. Conclusions

In this work, an offline RP × RP comprehensive two-dimensional liquid chromatography method with different pH values is evaluated for the analysis of alkaloids from* C. yanhusuo* extract. The first-dimension separation is performed using a base-resistant column with pH 10.5 using ammonium hydroxide in water and methanol. The second-dimension separation is carried out using a positive charge surface column with formic acid in acetonitrile and water as mobile phase. The orthogonality of the two dimensions is significantly improved by using different pH values. The effective peak capacity in the first dimension is up to 94 and is 205 for the second dimension, respectively. Two-dimensional separation provided excellent separation for the alkaloids from* C. yanhusuo* extract with an overall peak capacity of 9090. Sixteen compounds are tentatively identified from the extract by MS/MS and UV spectra. The developed method could be applied to the separation of complex alkaloid extract, especially for the discovering of coeluent alkaloids in conventional one-dimensional analysis.

## Figures and Tables

**Table 1:** Identified alkaloids in *C. yanhusuo* with MS/MS and UV spectra.

Number	*t*_*R*1_ (min) pH 10.5	*t*_*R*2_ (min) pH 3.0	*λ*_PDA_ (nm)	[M+H]^+^ (*m*/*z*)	Fragment ions (*m*/*z*)	Identified compound
1	13.05	19.01	233, 346	330.3	269.3, 251.7	Tetrahydroprotopapaverine [35]
2	13.55	8.50	229, 265	352.2	337.4, 322.4	13-Methyl-dehydrocorydalmine [36]
3	15.45	2.04	230, 341	366.2	333.9, 279.0	Dehydrocorydaline [37]
4	16.34	6.89	231, 262	328.2	313.0, 279.4	Demethylcorydalmine [35]
5	17.08	14.02	234, 276	320.2	292.3, 275.1	Coptisine [38]
6	22.32	15.48	217, 282	338.2	322.2, 294.3	Columbamine [39]
7	22.60	7.52	242, 273	342.2	278.0, 178.3	Tetrahydrocolumbamine [40]
8	22.88	5.26	224, 282	352.2	306.2, 293.2	13-Methylpalmatrubine [41]
9	24.39	4.73	214, 288	354.2	336.2, 275.2	Protopine [42]
10	25.26	5.41	222, 276	356.2	310.2, 294.0	D-Glaucine [43]
11	26.81	3.53	260, 282	352.2	336.2, 308.2	Dehydrocorybulbine [44]
12	27.06	5.26	220, 280	356.2	191.9, 165.2	D,L-Tetrahydropalmatine [44]
13	28.09	19.01	226, 346	352.2	337.4, 308.2	Palmatine [44]
14	29.16	15.48	220, 266	340.2	324.0, 292.0	Canadine [40]
15	30.18	9.80	245, 374	324.2	307.2, 248.8	D,L-Tetrahydrocoptisine [44]
16	31.64	22.02	209, 285	370.2	354.4, 295.5	Fumaricine [40]
